# Role of immune cell infiltration and small molecule drugs in adhesive capsulitis: Novel exploration based on bioinformatics analyses

**DOI:** 10.3389/fimmu.2023.1075395

**Published:** 2023-02-09

**Authors:** Hailong Liu, Baoxi Yu, Zengfa Deng, Hang Zhao, Anyu Zeng, Ruiyun Li, Ming Fu

**Affiliations:** ^1^ Department of Joint Surgery, The First Affiliated Hospital of Sun Yat-sen University, Guangzhou, Guangdong, China; ^2^ Guangdong Provincial Key Laboratory of Orthopedics and Traumatology, The First Affiliated Hospital of Sun Yat-sen University, Guangzhou, Guangdong, China; ^3^ China-Japan Friendship School of Clinical Medicine, Peking University, Beijing, China; ^4^ Department of Anesthesiology, Guangdong Provincial People’s Hospital, Guangdong Academy of Medical Sciences, Guangzhou, Guangdong, China

**Keywords:** adhesive capsulitis, frozen shoulder, immune infiltration, CIBERSORTx, bioinformatics, shoulder capsule

## Abstract

**Background:**

Adhesive capsulitis (AC) is a type of arthritis that causes shoulder joint pain, stiffness, and limited mobility. The pathogenesis of AC is still controversial. This study aims to explore the role of immune related factors in the occurrence and development of AC.

**Methods:**

The AC dataset was downloaded from Gene Expression Omnibus (GEO) data repository. Differentially expressed immune-related genes (DEIRGs) were obtained based on R package “DESeq2” and Immport database. Gene Ontology (GO) and Kyoto Encyclopedia of Genes and Genomes (KEGG) were performed to explore the functional correlation of DEIRGs. MCC method and Least Absolute Shrinkage and Selection Operator (LASSO) regression were conducted to identify the hub genes. The immune cell infiltration in shoulder joint capsule between AC and control was evaluated by CIBERSORTx, and the relationship between hub genes and infiltrating immune cells was analyzed by Spearman’s rank correlation. Finally, potential small molecule drugs for AC were screened by the Connectivity Map database (CMap) and further verified by molecular docking.

**Results:**

A total of 137 DEIRGs and eight significantly different types of infiltrating immune cells (M0 macrophages, M1 macrophages, regulatory T cells, Tfh cells, monocytes, activated NK cells, memory resting CD4+T cells and resting dendritic cells) were screened between AC and control tissues. MMP9, FOS, SOCS3, and EGF were identified as potential targets for AC. MMP9 was negatively correlated with memory resting CD4+T cells and activated NK cells, but positively correlated with M0 macrophages. SOCS3 was positively correlated with M1 macrophages. FOS was positively correlated with M1 macrophages. EGF was positively correlated with monocytes. Additionally, dactolisib (ranked first) was identified as a potential small-molecule drug for the targeted therapy of AC.

**Conclusions:**

This is the first study on immune cell infiltration analysis in AC, and these findings may provide a new idea for the diagnosis and treatment of AC.

## Introduction

Adhesive capsulitis (AC), also known as frozen shoulder, is a common shoulder disease. The main clinical manifestations of AC are pain, stiffness, and gradual loss of both active and passive activities of the affected shoulder resulting from progressive fibrosis and contracture of the glenohumeral capsule (1). The prevalence of AC in the general population is 2% to 5%, and a majority of patients are middle-aged women aged 40-70 years (2–4). Traditionally, AC is considered a self-limited disease, which is usually relieved completely within 1-2 years. However, several studies have reported that 20% to 50% of patients may suffer from long-term symptoms (2, 5–7). The causes of the disease are still unclear. Some mainstream academic views have linked its occurrence and development to the following factors: inflammatory reaction (8–11), fibrous tissue hyperplasia (12–15), immune factors (14, 16, 17), endocrine factors (18–20), and vascular factors (16, 21–23). Therefore, identifying the biomarkers and revealing the potential mechanism of AC is the key to the early treatment of AC.

Immune cells play an important role in the occurrence and development of many diseases. AC also contains some immune components, such as macrophages, B lymphocytes, and mast cells (24). Previous studies have shown that the pathological process of AC begins with an immune response, which then progressively worsens inflammation and eventually leads to fibrosis of the shoulder capsule (16, 17). Moeed et al. (25)confirmed the role of IL-17A driven AC pathogenesis and revealed the immune landscape of AC from the immune system dominated by macrophages to the immune system rich in T cells. However, the immune mechanisms of AC in the shoulder capsule have not been investigated thoroughly. Therefore, a systematic and effective method is urgently needed to identify immune-related genes and assess the contribution of immune cells in AC.

With the rapid development of RNA sequencing technology, bioinformatics analysis can be applied to identify key genes and biomarkers for many diseases, as well as to differentiate immune cell types (26). CIBERSORTx is a popular analytical tool to estimate the abundances of 22 immune cell types in a mixed cell population, using gene expression data (27). Hence, it can help us analyze the composition of immune cells in AC.

**Figure 1:**
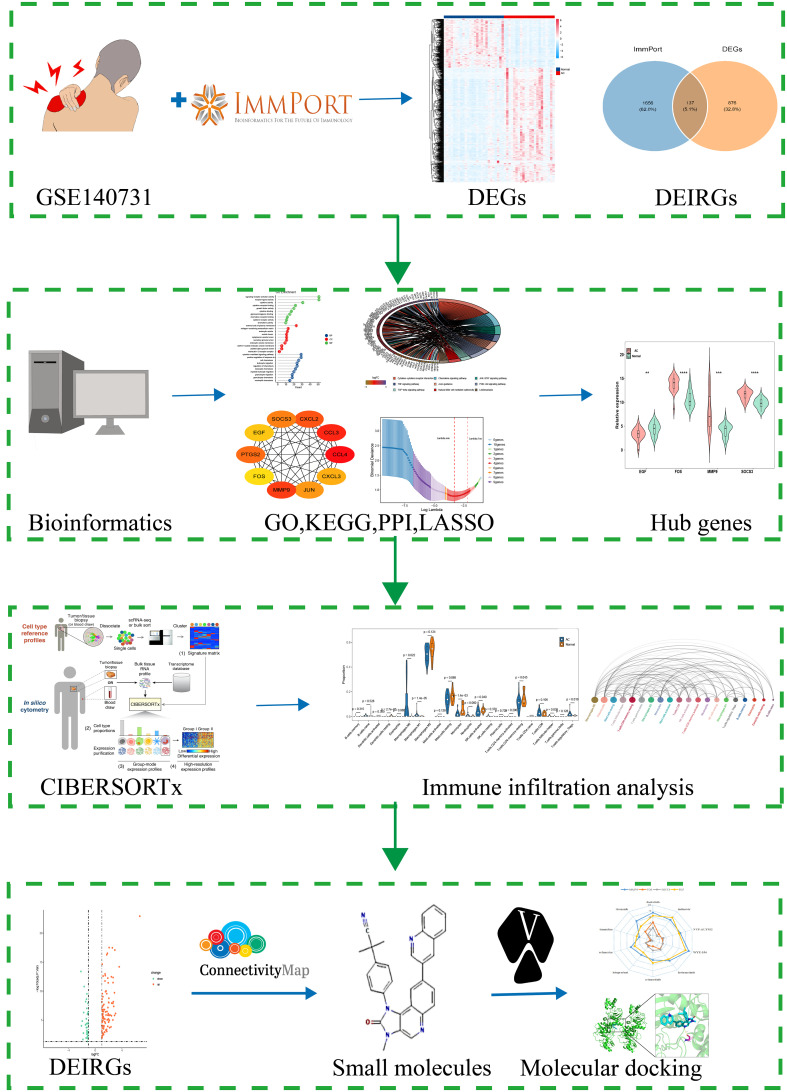
Flow chart of this study. **p<0.01,***p<0.001 and ****p<0.0001.

In this study, we used CIBERSORTx for the first time to evaluate the immune cell infiltration in AC. R package “DESeq2” was conducted to screen differential expressed genes (DEGs). PPI and LASSO logistic regression were used to identify hub genes. Wilcoxon test was conducted to identify the significant differences of immune cells in AC and control. Moreover, we explored the association between the hub genes and the infiltrating immune cells, providing the cornerstone for future study in this area. More importantly, the potential small molecule drugs targeted AC were carried out by using the CMap database and the verification of the potential mechanism was conducted by molecular docking. This study not only systematically analyzed the infiltration of immune cells in the shoulder capsule of AC, but also identified immune-related key genes and possible small molecule drugs for AC. The findings of this study will provide new perspectives on the early diagnosis and treatment of AC. The flow chart of this study is showed in **Figure 1**.

## Materials and methods

### Data download and preprocessing

Gene expression profiling of AC was downloaded from Gene Expression Omnibus (GEO) data repository (https://www.ncbi.nlm.nih.gov/geo/). The search strategy [adhesive capsulitis (All Fields) OR frozen shoulder (All Fields)] and [“Homo sapiens” (Organism)] was adopted. GSE140731 was used in our study, which contained a total of 48 samples (28). Gene annotation file (GENCODE - Human Release 40) was downloaded from GENCODE (https://www.gencodegenes.org) and used to convert “probe id” to “symbol” in the expression matrix. For multiple probes corresponding to the same gene symbol, we calculated the maximum value as its expression level. All analyses in this study were performed using the R software version (4.1.2).

### Immune-related genes collection

A total of 1,793 non-duplicated immune-related genes (IRGs) were obtained from the ImmPort database (29), which is one of the largest subject level open repositories of human immunology data and funded by the National Institutes of Health (NIH), National Institute of Allergy and Infectious Diseases (NIAID), Division of Allergy, Immunology, and Transplantation (DAIT).

### Identification of differentially expressed genes (DEGs) and differential expressed immune-related genes (DEIRGS)

R package “DEseq2” was used to screen for differential expressed genes (DEGs). The select criteria of DEGs were set as adjusted p-value < 0.05 and |log_2_ fold change| > 1. To obtain differentially expressed immune-related genes (DEIRGS), the DEGs were overlapped with the above 1,793 IRGs. Heat map, volcano plots and venn diagram created by “ggplot2” package were used for visualization.

### GO, KEGG, PPI network analysis and hub genes screening

Gene Ontology (GO), which involved biological processes (BP), cellular components (CC) and molecular functions (MF), and Kyoto Encyclopedia of Genes and Genomes (KEGG) pathway enrichment analysis were conducted by using “clusterProfiler” package, and the threshold values of p and q were set as 0.05. The results were visualized by R packages “ggpubr”, “ggplot2”, and “Goplot”. STRING (version11.5, https://cn.string-db.org/) was used to construct the protein-protein interaction (PPI) network with a confidence score >0.4. Cytoscape (version3.9.1) was used for visualization and the plug-in cytoHubba was used to calculate the ranking of DEIRGs. We selected the top 10 genes of the MCC method. Then, based on the 10 genes, we used the Least Absolute Shrinkage and Selection Operator (LASSO) Cox regression analysis by using the R package”glmnet” to identify the best hub genes.

### Assessment of immune cell infiltration

CIBERSORTx (https://cibersortx.stanford.edu/) is an analytical tool to provide an estimation of the abundances of infiltrating immune cell types in a mixed cell population, using a normalized gene expression matrix. The raw counts of the gene expression matrix were converted into TPM (transcripts per million) in R software before being submitted. The immune infiltration analyses were performed with 1000 permutations and the LM22 was adopted as a reference gene expression signature. The LM22 dataset contains 547 signature genes that distinguish between 22 human immune cell phenotypes (27). Only samples with a CIBERSORTx p value < 0.05 was filtered and selected for the subsequent analysis. R packages “ggplot2”, “ggraph”, and “corrplot” were used for visualization.

### Correlation analysis between hub genes and infiltrating immune cells

The relationship of the 4 hub genes with the levels of infiltrating immune cells was explored using Spearman’s rank correlation analysis in R software. The results were visualized using the R package “ggplot2”.

### Identification of candidate small molecule compounds

The Connectivity Map (30), or CMap, is a resource that uses cellular responses to perturbation to find relationships between diseases, genes, and therapeutics. It could be used to predict potential small molecules that affect that phenotype caused by specific gene expression. To explore the potential small molecule drugs that may treat AC, the 137 DEIRGs mentioned previously were divided into two groups (up-regulation down-regulation) and then submitted to the CMap database. A negative enrichment score indicates that small molecules may reverse the expression of the genes and have potential therapeutic value.

### Molecular docking verification

Molecular docking between hub genes and small molecule compounds was carried out to predict the accuracy of the pivotal components and prediction targets using AutoDock Vina (v1.5.7). PubChem database (https://pubchem.ncbi.nlm.nih.gov/), RCSB protein data (http://www.rcsb.org/), and PDBe-KB database (https://www.ebi.ac.uk/pdbe/pdbe-kb/) were selected to download the MOL2 format of ligands and PDB format of proteins. Crystal of proteins was introduced to Pymol software (https://pymol.org/2/; version 2.4.1) to conduct dehydration and separation of ligands. Subsequently, the crystal conducted was introduced to AutoDockTools to build a docking grid box of targets. Molecular dockings were achieved *via* AutoDock Vina. The lower affinity scores, one of the results of molecular docking, represent a more stable binding affinity of protein and ligand. Eventually, the complexes of protein and compound were visualized by Pymol software.

## Results

### Identification of DEGs and DEIRGs

**Figure 2:**
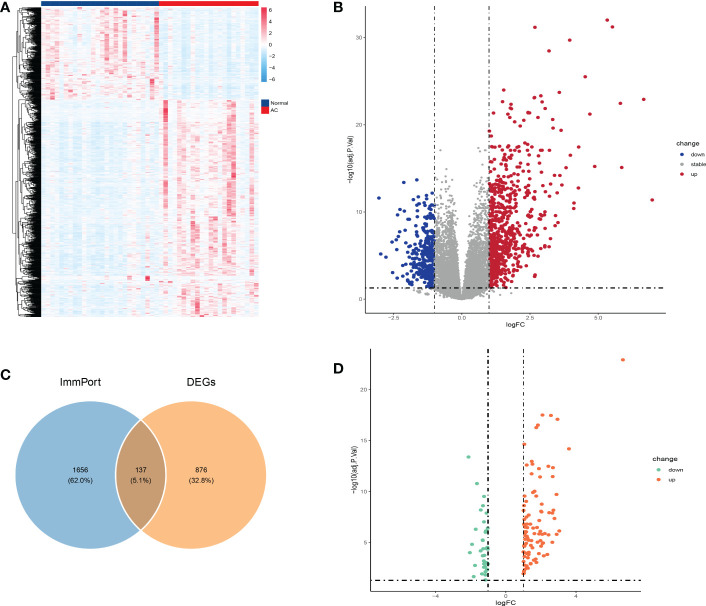
Identification of DEGs and DEIRGs. DEGs were visualized by heatmap **(A)** and volcano map **(B)**. Venn diagram was used to visualize the acquisition of 137 DEIRGs **(C)**. DEIRGs contain 99 up-regulated genes and 38 down-regulated genes **(D)**.

The GSE140731 dataset was platform-based on GPL24676 and contained a total of 48 samples, including 22 AC samples and 26 control samples. We used the R package “DESeq2” to identify 1,012 DEGs (698 up-regulated and 314 down-regulated) from the dataset, as shown in the heatmap and volcano map **(** **Figures 2A, B** **).** Next, to obtain DEIRGs, the intersection of DEGs with immune-related genes from the Immport database was visualized by venn diagram **(** **Figure 2C** **)**. Finally, 137 genes were screened, including 99 up-regulated and 38 down-regulated **(** **Figure 2D** **)**.

### GO and KEGG enrichment analysis of DEIRGs

**Figure 3:**
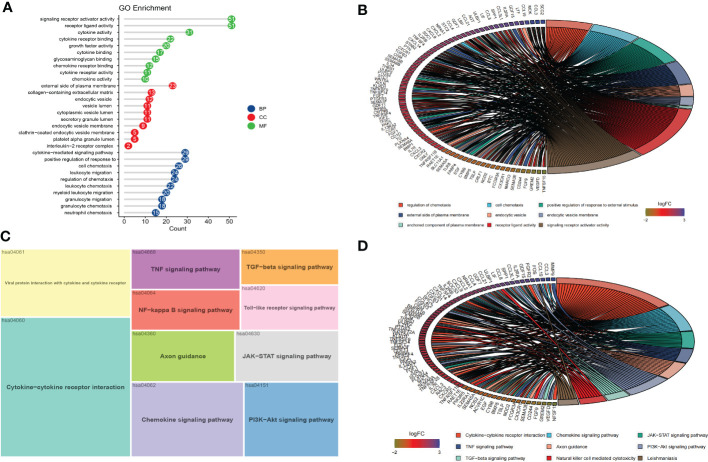
Functional enrichment and PPI of DEIRGs. **(A, B)** The results of GO analysis were displayed in lollipop and circle charts. **(C, D)** The results of KEGG analysis were displayed in treemap and circle charts.

To explore the potential biological functions and signaling pathways of DEIRGs, we performed GO and KEGG enrichment analysis with R package “clusterProfiler”. The GO analysis results revealed that the DEIRGs were mostly enriched in positive regulation of response to external stimulus, cytokine-mediated signaling pathway, cell chemotaxis, regulation of chemotaxis and leukocyte migration for biology process (BP); external side of plasma membrane, collagen-containing extracellular matrix, endocytic vesicle, secretory granule lumen and cytoplasmic vesicle lumen for cellular component (CC); receptor ligand activity, signaling receptor activator activity, cytokine activity, cytokine receptor binding and growth factor activity for molecular function (MF) **(** **Figures 3A, B** **)**. Otherwise, KEGG pathway enrichment analysis showed that pathways were mainly associated with cytokine-cytokine receptor interaction, viral protein interaction with cytokine and cytokine receptor, chemokine signaling pathway, PI3K-Akt signaling pathway, and JAK-STAT signaling pathway **(** **Figures 3C, D** **)**.

### PPI, LASSO analysis and hub genes identification

**Figure 4:**
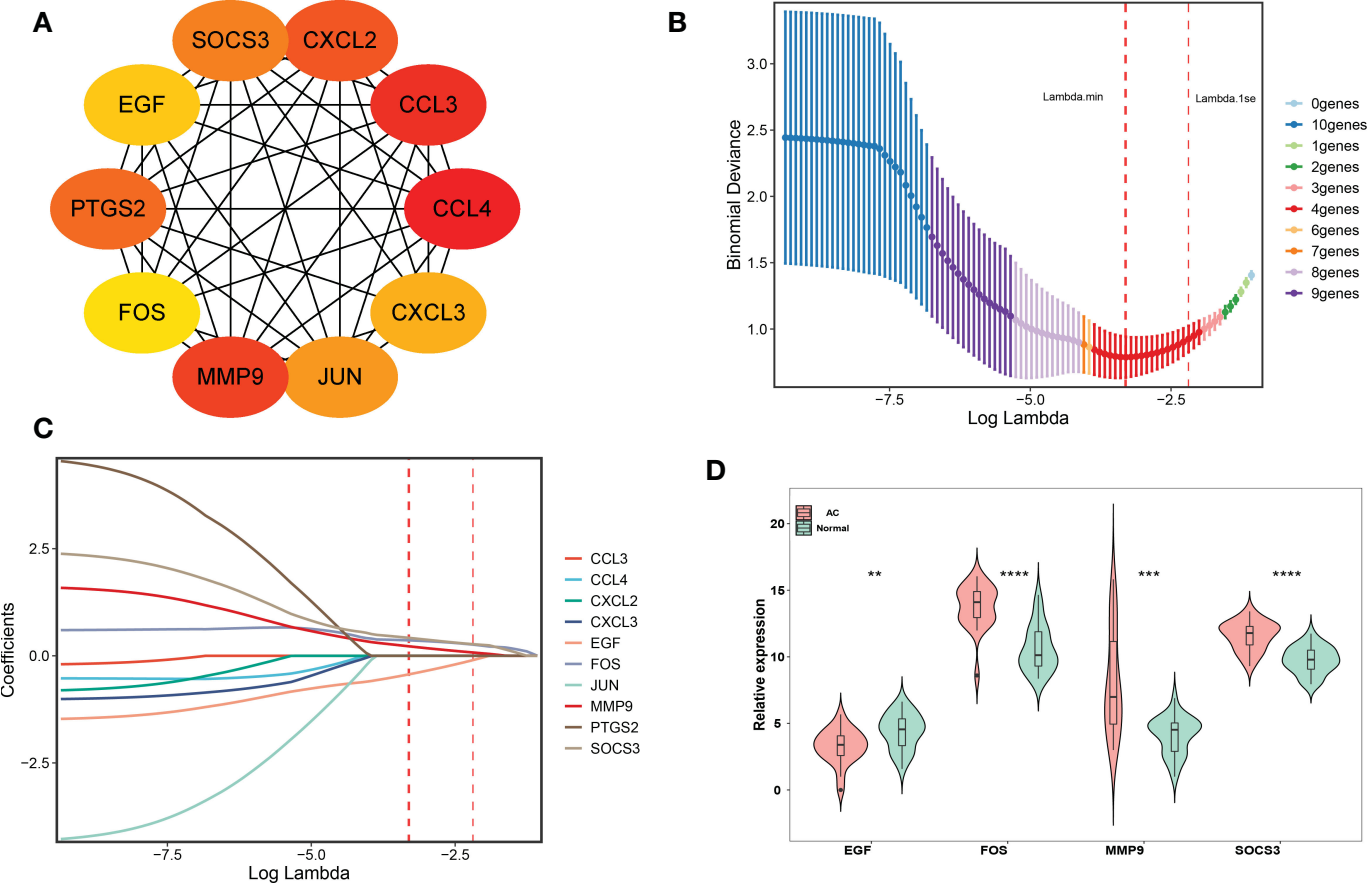
PPI network establishment and hub genes identification. **(A)** Top 10 genes based on MCC method, and yellow to red represent progressively higher scores. **(B, C)** LASSO logistic regression was performed to further identify hub genes. The red dotted line on the left represents lambda.min, and the red dotted line on the right represents lambda.1se. **(D)** The final 4 hub genes were shown in the violin diagram. ^∗∗^ *p* < 0.01,^∗∗∗^p < 0.001 and ^∗∗∗∗^ *p* < 0.0001.

Two different algorithms, namely, PPI and LASSO regression, were used to identify the hub genes of DEIRGs. We obtained the PPI results from the STRING database and then used the CytoHubba plugin MCC to calculate the score of each node gene. The top 10 genes (CCL4, CCL3, MMP9, CXCL2, PTGS2, SOCS3, JUN, CXCL3, EGF, FOS) of the MCC method are shown in **Figure 4A**. Finally, 4 hub genes (MMP9, SOCS3, EGF, FOS) were identified using the least absolute shrinkage and selection operator (LASSO) regression algorithm **(** **Figures 4B–D** **)**.

### Immune cell infiltration in AC tissue

**Figure 5:**
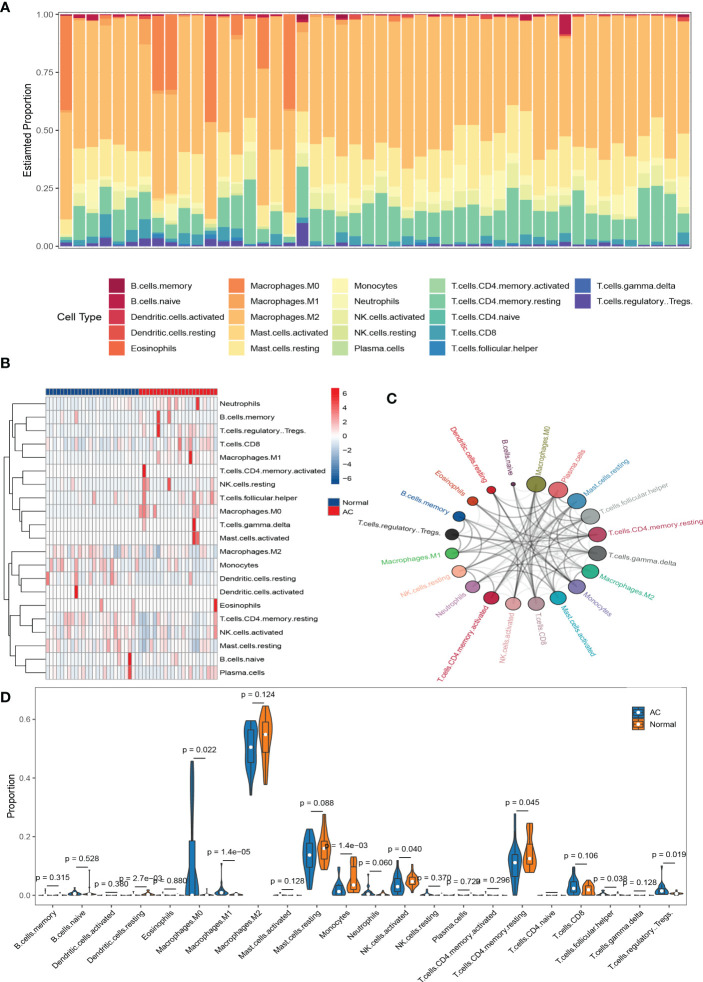
The results of immune cell infiltration analysis. Composition of 22 types of infiltrating immune cells in each sample was shown in a bar chart **(A)** and a heat map**(B)**. Network diagram of 22 types of infiltrating immune cells. Larger circles represent stronger interactions with other immune cells **(C)**. Wilcoxon test was used to identify significantly different infiltrating immune cells in AC and normal tissues **(D)**.

The results of immune cell infiltration were downloaded from the CIBERSORTx website and visualized by R software. First, a bar chart **(** **Figure 5A** **)** and a heat map **(** **Figure 5B** **)** were used to show the composition of 22 kinds of immune cells in each sample. In the bar chart, the color represents the estimated proportion of different immune cells in each sample, and the sum of the total proportion is 1. The heat map represents the difference of immune cell abundance between AC samples and control samples. The results showed that M2 macrophages, activated NK cells, resting mast cells, Monocytes, and memory resting CD4+T cells were the main infiltration immune cells. After removing the immune cells with an expression abundance of 0 in all samples, we evaluated the correlation of the remaining 20 kinds of infiltration immune cells in AC shoulder capsule tissues. The correlation map **(** **Figure S1, A** **)** showed that M2 macrophages were negatively correlated with M0 macrophages (r = -0.43) and regulatory T cells (r = -0.68), positively correlated with resting mast cells (r =0.58). Activated NK cells were strongly positively associated with memory resting CD4+T cells (r = 0.87), eosinophils (r = 0.59), plasma cells (r =0.57) and resting mast cells (r = 0.45), but substantially negatively associated with resting NK cells (r = -0.49) and M0 macrophages (r = -0.59). Resting mast cells were strongly positively connected with M2 macrophages (r = 0.58), activated NK cells (r = 0.45), memory resting CD4+T cells (r = 0.43), but substantially negatively correlated with M0 macrophages (r = -0.8), resting NK cells (r = -0.46), Gamma delta T cells (r = -0.43), Tfh cells (r = -0.43), memory activated CD4+T cells (r= -0.44) and active mast cells (r = -0.48). Monocytes were strongly positively connected with naïve B cells (r = 0.64), but negatively correlated with M0 macrophages (r = -0.53). Memory resting CD4+T cells were strongly positively associated with plasma cells (r = 0.46), activated NK cells (r = 0.87), resting mast cells (r = 0.43) and eosinophils (r = 0.59), negatively correlated with M0 macrophages (r = -0.73). The network diagram **(** **Figure 5C** **)** showed that M0 macrophages, plasma cells, and resting mast cells were closely related to other infiltrating immune cells, but naïve B cells and resting dendritic cells were weakly related to other infiltrating immune cells. Otherwise, based on the composition of infiltrating immune cells in shoulder capsule tissues, we could completely distinguish AC from normal tissues by PCA analysis (**Figure S1, B** **)**. As shown in the violin diagram **(** **Figure 5D** **),** the degree of M0 macrophages, M1 macrophages, regulatory T cells and Tfh cells infiltration in AC tissues were significantly higher than in normal tissues (p < 0.05), but the degree of monocytes, activated NK cells, memory resting CD4+T cells and resting dendritic cells infiltration were significantly lower than in normal tissues (p < 0.05).

### Correlation between hub genes and differential infiltrating immune cells in AC

**Figure 6:**
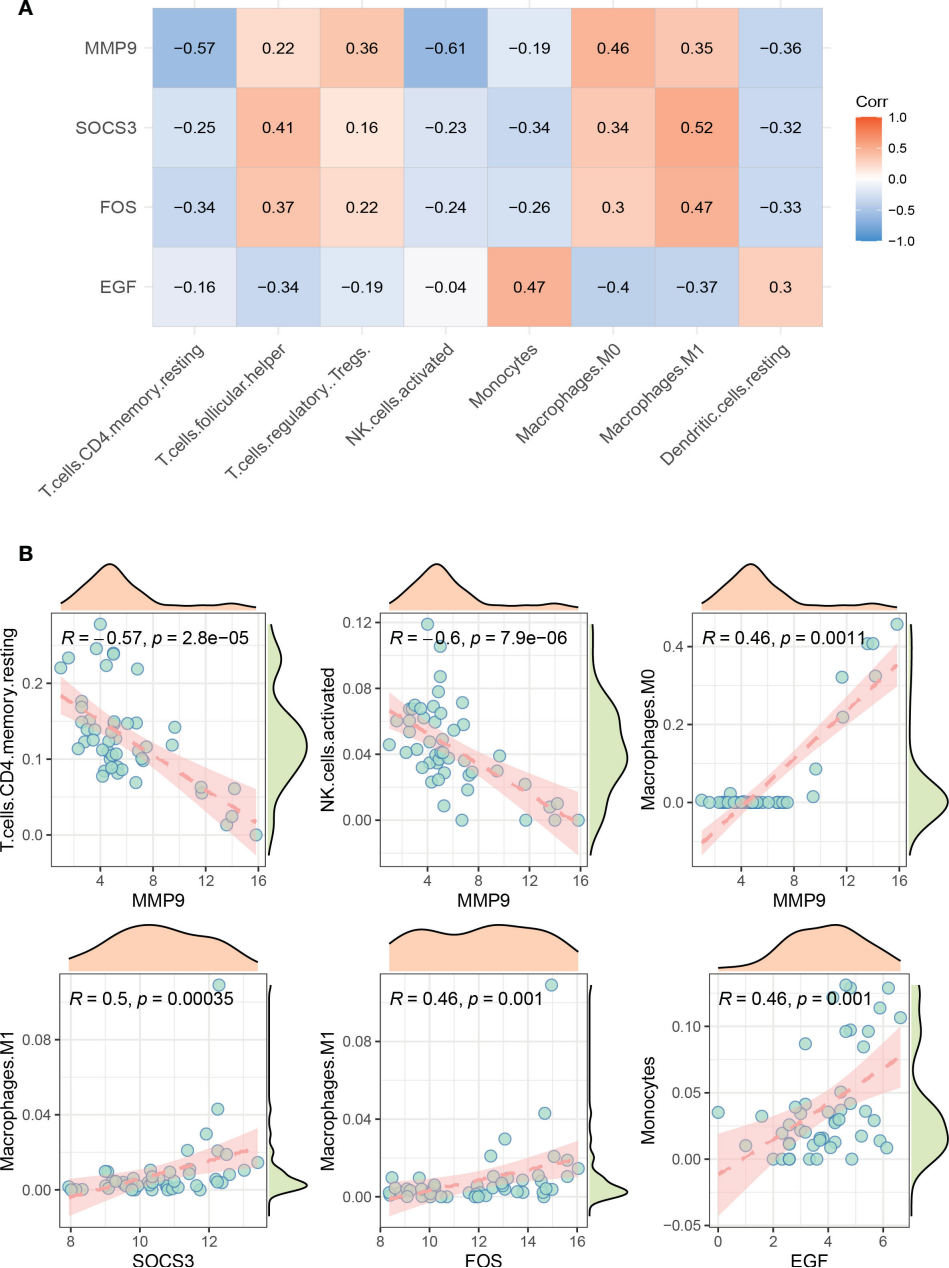
Visualization of correlation between hub genes and infiltrating immune cells. **(A)** Correlation between 4 hub genes and 8 significantly different immune cells. Red represents positive correlation, blue represents negative correlation. **(B)** Significantly correlated hub genes and immune cells were screened by adjusted p-value < 0.05.

We analyzed the correlation of 4 hub genes (MMP9, SOCS3, EGF, FOS) with 8 significantly differential infiltrating immune cells in AC. The results are presented in **Figure 6A**. Significantly correlated hub genes and immune cells were screened by adjusted p-value < 0.05. As shown in **Figure 6B**, MMP9 was negatively correlated with memory resting CD4+T cells (r = -0.57, p = 2.8e-05) and activated NK cells (r = -0.6, p = 7.9e-06), but positively correlated with M0 macrophages (r = 0.46, p = 0.0011). SOCS3 was positively correlated with M1 macrophages (r = 0.5, p = 0.00035). FOS was positively correlated with M1 macrophages (r = 0.46, p = 0.001). EGF was positively correlated with monocytes (r = 0.46, p = 0.001).

### Small molecule drugs screening and molecular docking

**Figure 7:**
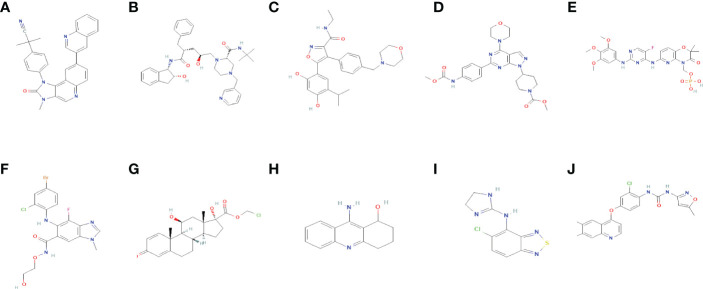
The 2D chemical structures of 10 small molecule drugs. **(A)** dactolisib **(B)** indinavir **(C)** NVP-AUY922 **(D)** WYE-354 **(E)** fostamatinib **(F)** selumetinib **(G)** loteprednol **(H)** velnacrine **(I)** tizanidine **(J)** tivozanib.

**Figure 8:**
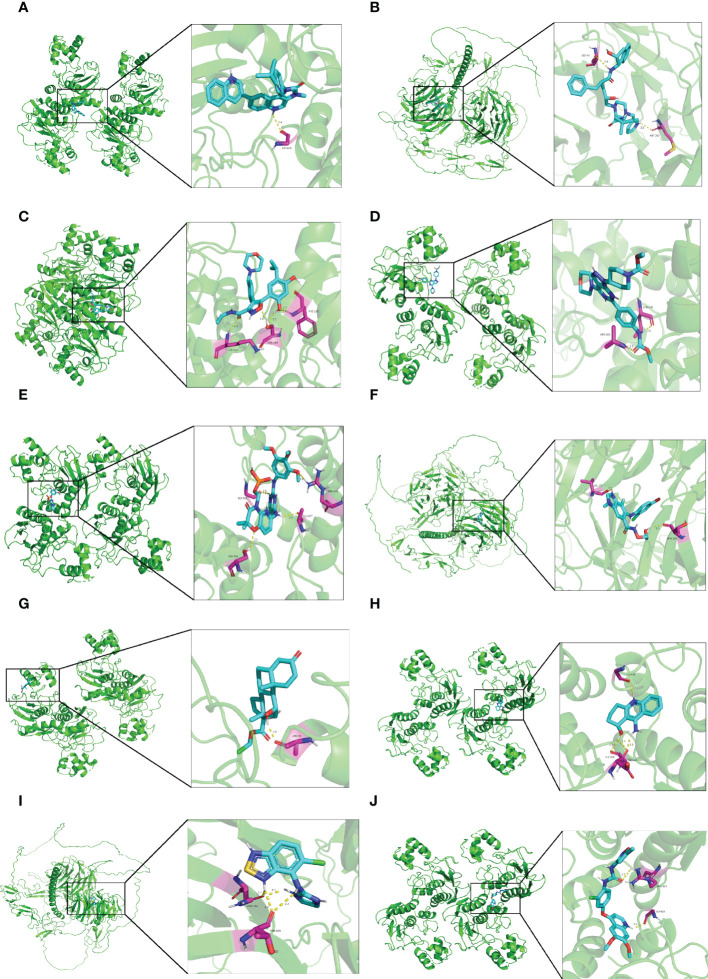
Molecular docking diagram of small molecule drugs and target genes. **(A)** dactolisib-MMP9 **(B)** indinavir-EGF **(C)** NVP-AUY922-MMP9 **(D)** WYE-354-MMP9 **(E)** fostamatinib-MMP9 **(F)** selumetinib-MMP9 **(G)** loteprednol-MMP9 **(H)** velnacrine-MMP9 **(I)** tizanidine-EGF **(J)** tivozanib-MMP9.

As shown in **Table 1**, the top 10 small molecule compounds (dactolisib, indinavir, NVP-AUY922, WYE-354, fostamatinib, selumetinib, loteprednol, velnacrine, tizanidine, tivozanib) with highest negative score were screened as potential drugs for AC. The 2D chemical structures downloaded from PubChem are presented in **Figures 7A–J**. Then, these 10 small molecule compounds were docked with screened 4 core targets (MMP9, SOCS3, EGF, FOS) by using AutoDock Vina software. The binding energy for molecular docking is presented in **Table 2**. We selected the lowest binding energy between each small molecule compound and the core target for visualization **(** **Figures 8A–J** **)**. The yellow dotted lines in the figure represent hydrogen bonds. For instance, dactolisib may play its biological role by binding to MMP9 and forming a hydrogen bond on the amino acid GLY-428 near the active site.

**Table 1:** 10 compounds with the highest negative enrichment score from Cmap database.

Rank	Score	Compound	Description
**1**	-88.89	dactolisib	MTOR inhibitor
**2**	-86.81	indinavir	HIV protease inhibitor
**3**	-85.29	NVP-AUY922	HSP inhibitor
**4**	-81.82	WYE-354	MTOR inhibitor
**5**	-81.62	fostamatinib	SYK inhibitor
**6**	-81.51	selumetinib	MEK inhibitor
**7**	-76.46	loteprednol	Glucocorticoid receptor agonist
**8**	-73	velnacrine	cholinesterase inhibitor
**9**	-72.92	tizanidine	Adrenergic receptor agonist
**10**	-71.34	tivozanib	VEGFR inhibitor

**Table 2:** Binding energy for molecular docking.

Targets Molecules	MMP9	FOS	SOCS3	EGF
**dactolisib**	-8.7	-7.1	-6.2	-8.2
**indinavir**	-8.3	-5.4	-5	-8.8
**NVP-AUY922**	-8.2	-5.4	-5.6	-8
**WYE-354**	-9.5	-5.5	-5.5	-9
**fostamatinib**	-8.1	-5.7	-5.8	-7.8
**selumetinib**	-7.2	-4.9	-5.6	-7.7
**loteprednol**	-6.9	-5	-5.2	-6.4
**velnacrine**	-7.4	-5.1	-5.1	-6.8
**tizanidine**	-6.1	-4.4	-4.1	-6.5
**tivozanib**	-8.1	-5.9	-5.3	-7

## Discussion

AC is a chronic shoulder disease characterized by pain, stiffness, and dysfunction. Although it is traditionally believed that most people’s symptoms can be completely relieved within 1-2 years, more and more clinical studies challenge this theory (2, 4, 6). At present, there are few studies on the molecular mechanism of AC, and there are still controversies about its pathogenesis. Therefore, the most effective treatment for AC is still uncertain. Immune factors play an important role in the diagnosis and treatment of many diseases, but little is known about their role in AC. In this study, we used a systematic and comprehensive bioinformatics method to explore the immune-related hub genes of AC, analyze the role of immune cell infiltration in the shoulder capsule, and predict potential small molecule drugs for AC.

We performed a comprehensive analysis of the GSE140731 dataset and identified a total of 137 DEIRGs, including 99 up-regulated and 38 down-regulated. GO enrichment analysis showed that the DEIRGs were associated with cytokine activity, chemokine activity, and cytokine-mediated signaling pathway. KEGG analysis indicated that these DEIRGs were primarily enriched in cytokine-cytokine receptor interaction, chemokine signaling pathway and natural killer cell mediated cytotoxicity. These results confirm previous findings that inflammation plays an important role in the development of AC and suggest that immune response may be involved, which is consistent with our goal. To improve the reliability of the results, we integrated PPI and LASSO to screen hub genes. Four genes were finally screened, namely MMP9, EGF, FOS, and SOCS3. Among them, only MMP9 has been reported in AC.

MMP9 is a member of the matrix metalloproteinase (MMP) family and its main function is to maintain the dynamic balance of extracellular matrix. As early as 2005, Blaine et al. (31) and Voloshin et al. (32) successively confirmed that patients with rotator cuff tears were prone to bursitis, and the expression of MMP9 in subacromial bursitis was significantly higher than that in the control group. Yi Wang et al. (33)found that targeted knockout of TNF-α can downregulate the expression of MMP9, thereby reducing the inflammatory response of bursitis. In 2019, a Brazilian study (34) pointed out that women carrying the allele of MMP9 would increase the risk of frozen shoulder. In the same year, a Korean study (35) confirmed that MMP9 was significantly overexpressed in frozen shoulder patients. These studies are consistent with our bioinformatics analysis results and indicate that MMP9 has potential value in the diagnosis and treatment of AC. Although there have been no studies on MMP9 regulating immune responses in AC, MMP9 has been shown to exert immune function in many other diseases (36–40). SOCS3 is a member of suppressor of cytokine signaling (SOCS) family. It is an important regulator of cytokine signal transduction and immune response. SOCS3-mediated m6A mRNA methylation can regulate T cell homeostasis (41). SOCS3 acts as a regulator of macrophage polarization, and its deficiency can skew macrophages toward an M1 phenotype (42). In IBD-related diseases, SOCS3 can regulate the expression and differentiation of T cells and B cells (43). FOS is one of the four members of the FOS gene family (FOS, FOSB, FOSL1, and FOSL2), which can form the transcription factor complex AP-1 and is considered a regulator of cell proliferation. Non-coding RNA can regulate immune response by targeting FOS (44, 45). And FOS can transcribe and activate the target gene NFATc1 and participate in the active immune response (46). Ryoko Yoshida et al. (47)confirmed that FOS can inhibit some innate and adaptive immune responses in dendritic cells. Epidermal growth factor (EGF) is a multifunctional growth factor. By combining with its receptor (EGFR), EGF can induce the growth and migration of tissue cells, promote the expression of differentiation genes, and maintain the normal metabolism of epithelial cells (48). In early life, EGF can promote the maturation of the immune system (49). Christina Groepper et al. (50)found that EGF signaling can be modified by HCV to exert antiviral immunity by upregulating CXCR2 expression. These studies provide some theoretical support for further exploring the immune function of these genes in AC.

To our knowledge, this is the first study about immune cell infiltration in AC tissue. Compared with control group, M0 macrophages, M1 macrophages, regulatory T cells, and Tfh cells were significantly higher in AC shoulder capsule tissues, while monocytes, activated NK cells, memory resting CD4+T cells, and resting dendritic cells were significantly lower in AC. Interestingly, although the infiltration proportions of M2 macrophages and resting mast cells were relatively high, the difference between AC and control groups was not statistically significant. Our results showed that M0 macrophages were significantly increased and mainly polarized into M1 macrophages in AC tissues, which may be an important reason for aggravating AC. Although the role of immune cells in AC has not been elucidated, their relationship with inflammation has been reported. During inflammation or tissue injury, pro-inflammatory mediators attract migrating monocytes to sites of inflammation and promote their differentiation toward macrophages to activate them (51). Regulatory T cells and Tfh cells may mediate the immune inflammatory response by promoting fibrogenesis and cytokine production (52, 53). Imbalance of dendritic cells leads to disturbance of immune homeostasis, which in turn causes abnormal inflammatory activation (54, 55). NK cells are a double-edged sword in the process of inflammation, which may be related to the activation of T cells and their recruitment by DCs (56). To further explore the important role of immune infiltrating cells and hub genes in AC, we calculated the correlation among them. The most intriguing result was that SOCS3 was positively correlated with M1 macrophages (r = 0.5, p = 0.00035). Yao Chun Wang et al. (57) found that SOCS3 could be activated by a notch signal, thus promoting the polarization of M1 macrophages. However, another study (42) suggested that the lack of SOCS3 could promote the polarization of M1 macrophages. Therefore, further experiments are needed to verify the relationship between immune cells and hub genes.

CMap is a database often used to explore potential therapeutic drugs for diseases (58). We uploaded 99 up-regulated DEIRGs and 38 down-regulated DEIRGs to the database and successfully screened the top ten compounds with negative scores, of which dactolisib was the first. Dactolisib is a dual ATP competitive PI3K and mTOR inhibitor, which has been proven to have certain effects on tumors (59, 60), inflammatory diseases (61), polycystic kidney disease (62), and Alzheimer’s disease (63). Meanwhile, we performed molecular docking to validate the binding of hub genes and small molecules. This provides the basis for future basic pharmacological experiments in AC.

This study has some limitations. First, only one dataset was used to screen DEGs, which was considered a possible constraint. Second, it lacks useful clinical information, including the duration of the disease, etc. Last, we only used bioinformatics methods, and more *in vitro* and *in vivo* experiments are needed in the future.

In conclusion, we not only screened four hub DEIRGs, but also analyzed the immune cell infiltration of AC for the first time. Meanwhile, the potential small molecule drugs were predicted. These findings provide a new idea to the study the pathogenesis of AC and we will further validate the above results through *in vivo* and *in vitro* experiments in future studies.

## Data availability statement

Publicly available datasets were analyzed in this study. This data can be found here: https://www.ncbi.nlm.nih.gov/geo/query/acc.cgi?acc=GSE140731.

## Author contributions

MF and RL designed the study. HL, BY, and ZD drafted the manuscript. HZ and AZ made a significant contribution to the acquisition and integration of the data. MF and RL reviewed and revised the manuscript. All authors contributed to the article and approved the submitted version.

## Supplementary material

Supplementary Figure 1
**(A)** The correlation of 20 types of infiltrating immune cells in AC tissues, showing only p< 0.05. **(B)** PCA analysis was conducted to classify infiltrating immune cells between AC and control shoulder joint tissues.
