# Supplementary figures and images for: Role of immune cell infiltration and small molecule drugs in adhesive capsulitis: Novel exploration based on bioinformatics analyses
Source: Front Immunol. 2023 Feb 9;14:1075395. doi: 10.3389/fimmu.2023.1075395 (PMC9976580; doi:10.3389/fimmu.2023.1075395)

A

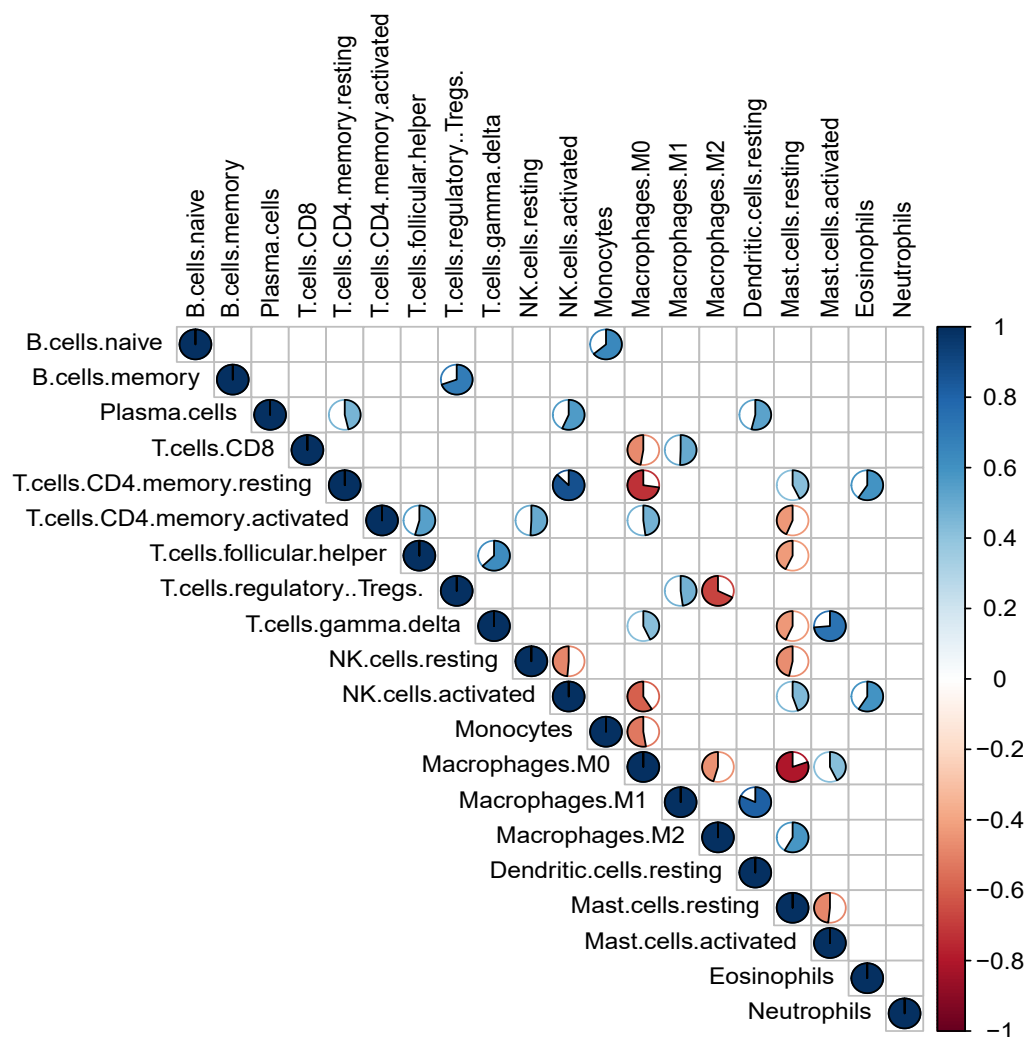

B

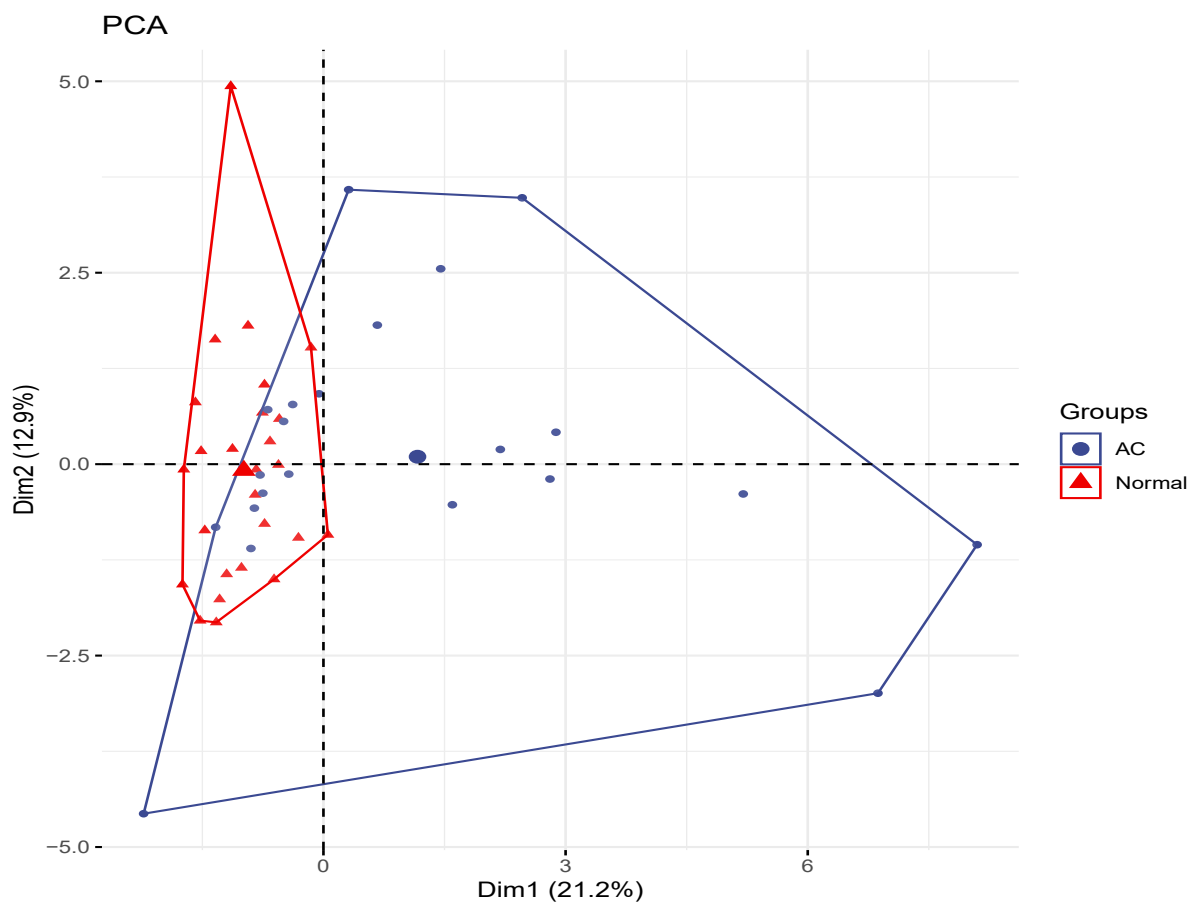

Supplement: Supplementary Figure 1 — (A) The correlation of 20 types of infiltrating immune cells in AC tissues, showing only p< 0.05. (B) PCA analysis was conducted to classify infiltrating immune cells between AC and control shoulder joint tissues. [file Image_1.pdf]
